# 30 A Qualitative Descriptive Exploration of the Experiences of Burn Therapists

**DOI:** 10.1093/jbcr/irae036.030

**Published:** 2024-04-17

**Authors:** Miranda L Yelvington, Rachel E Wood, Tyler Corson, Jiale Hu, Stacey Reynolds

**Affiliations:** Arkansas Children's Hospital, Little Rock, Arkansas; Virginia Commonwealth University, Richmond, Virginia; Virginia Commonwealth University, Ormond Beach, Florida; Department of Nurse Anesthesia/Virginia Commonwealth University, Richmond, Virginia; Arkansas Children's Hospital, Little Rock, Arkansas; Virginia Commonwealth University, Richmond, Virginia; Virginia Commonwealth University, Ormond Beach, Florida; Department of Nurse Anesthesia/Virginia Commonwealth University, Richmond, Virginia; Arkansas Children's Hospital, Little Rock, Arkansas; Virginia Commonwealth University, Richmond, Virginia; Virginia Commonwealth University, Ormond Beach, Florida; Department of Nurse Anesthesia/Virginia Commonwealth University, Richmond, Virginia; Arkansas Children's Hospital, Little Rock, Arkansas; Virginia Commonwealth University, Richmond, Virginia; Virginia Commonwealth University, Ormond Beach, Florida; Department of Nurse Anesthesia/Virginia Commonwealth University, Richmond, Virginia; Arkansas Children's Hospital, Little Rock, Arkansas; Virginia Commonwealth University, Richmond, Virginia; Virginia Commonwealth University, Ormond Beach, Florida; Department of Nurse Anesthesia/Virginia Commonwealth University, Richmond, Virginia

## Abstract

**Introduction:**

Exposure to patients who have experienced major traumatic events places burn therapists at risk for developing high levels of anxiety and stress, directly impacting their quality of life. Poor professional quality of life has been associated with negative physical and emotional characteristics in healthcare professionals and can subsequently influence patient safety and satisfaction, job retention, and productivity. The purpose of this study was to understand and describe the experiences of occupational and physical therapists who provide acute therapy to people who have sustained burn injuries.

**Methods:**

The study utilized a qualitative descriptive approach. Participants were selected using a maximum variation sampling strategy. Years of burn experience ranged from 2 to 37 years, and participants practiced in adult (40%), pediatric (20%), or mixed burn care (40%). Participating therapists represented all five American Burn Association Regions. Semi-structured interviews were completed with 10 participants (8 female, 7 physical therapists) to explore their experiences in their own words. Conventional content analysis was used to analyze the data collected from semi-structured interviews.

**Results:**

Six themes emerged after content analysis. These themes were: 1) the importance of therapeutic relationships to patient successes and therapists’ retention; 2) the autonomy and flexibility of burn therapists; 3) the impact of career longevity on compassion; 4) the uniqueness of burn team relationships and camaraderie; 5) the challenges of operating within the business of a healthcare system; and 6) the physical, mental and emotional challenges to the burn therapists’ resiliency.

**Conclusions:**

The overarching themes reflected the importance of therapeutic relationships to patient successes and therapists’ retention. The autonomy and flexibility of burn therapists were found to be a driver of professional quality of life as therapists were empowered by their ability to lead patient change. Therapists endorsed developing boundaries and methods to decrease the emotional impact of patient losses as their career progresses. Participants spoke about the positive and negative impact of being part of a multidisciplinary team, often feeling limited by the challenges inherent in operating within the business of a healthcare system.

**Applicability of Research to Practice:**

This research brings to light the physical, mental, and emotional challenges that impact burn therapists’ resiliency and which should be considered when creating programs to increase retention and professional quality of life in this population.

